# Anthracycline Induced Cardiac Disorders in Childhood Acute Lymphoblastic Leukemia: A Single-Centre, Retrospective, Observational Study

**DOI:** 10.3389/fphar.2021.598708

**Published:** 2021-03-29

**Authors:** Hui Yu, Yining Qiu, Hui Yu, Zhujun Wang, Jiawei Xu, Yun Peng, Xia Wan, Xiaoyan Wu, Runming Jin, Fen Zhou

**Affiliations:** Department of Pediatrics, Union Hospital, Tongji Medical College, Huazhong University of Science and Technology, Wuhan, China

**Keywords:** cardiac disorders, daunorubicin, chemotherapy, childhood, acute lymphoblastic leukemia

## Abstract

Anthracycline-associated cardiotoxicity is frequently seen in cancer survivors years after treatment, but it is rare in patients on chemotherapy. This study aimed to investigate the clinical characteristics of cardiac disorders in children with acute lymphoblastic leukemia (ALL) during chemotherapy. A retrospective case study was conducted in children with ALL, for whom electrocardiogram (ECG) and echocardiography (Echo) were regularly assessed before each course of chemotherapy. The cardiac disorders were diagnosed according to the Common Terminology Criteria for Adverse Events (CTCAE) Version 5.0. Binary logistic regression analysis was used to identify risk factors associated with cardiac disorders. There were 171 children eligible for the study, and 78 patients (45.61%) were confirmed as having cardiac disorders. The incidence of cardiac disorders was dependent upon the cumulative dose of daunorubicin (DNR) (*p* = 0.030, OR = 1.553, 95% CI: 1.005–3.108). Four patients (2.34%) presented with palpitation, chest pain, and persistent tachycardia, and they were cured or improved after medical intervention. A total of 74 patients (43.27%) had subclinical cardiac disorders confirmed by ECG or Echo. ECG abnormalities were commonly seen in the induction and continuation treatments, including arrhythmias (26, 15.20%), ST changes (24, 14.04%) and conduction disorders (4, 2.34%). Pericardial effusion (14, 8.19%), left ventricular hypertrophy (11, 6.43%), a widened pulmonary artery (5, 2.92%) and valvular insufficiency (5, 2.92%) suggested by Echo occurred after induction chemotherapy. Therefore, cardiac disorders with clinical manifestations are rare and need early intervention. Subclinical cardiac disorders are common but very hidden in children during ALL chemotherapy. Regular ECG and Echo could help paediatricians to identify and monitor patients with asymptomatic cardiac disorders earlier.

## Introduction

Acute lymphoblastic leukemia is the most common malignancy in children and currently chemotherapy is still the main treatment (Steliarova-Foucher et al., 2017). Anthracycline is a first-line chemotherapy drug with significant efficacy, increasing the five-year survival rate of children with leukemia from 30% in the 1960s to over 80% at present (Robison et al., 2009). However, cardiotoxicity is a well-recognized side effect that may limit the clinical use of anthracycline and even affect the quality of life and survival of patients with leukemia, although the morbidity of heart failure (HF) is less than 7.5% (Franco et al., 2011). According to the time of onset, anthracycline-induced cardiotoxicity is categorized as three types: sub-acute, acute, and chronic. Sub-acute and acute cardiotoxicity occur between the time of chemotherapy initiation to up to two weeks after completion of treatment, which usually manifest as conduction disorders or arrhythmias, rarely pericarditis or acute left HF (Dolci et al., 2008; Chinese Society of Clinical Oncology, Chinese Sopciety of Hematology, 2013). Chronic cardiotoxicity is further divided into early cardiotoxicity (onset within one year of chemotherapy) and late (onset after one year of chemotherapy), which mainly manifest as delayed arrhythmia, cardiomyopathy, or congestive heart failure (CHF) (Dolci et al., 2008; Chinese Society of Clinical Oncology, Chinese Sopciety of Hematology, 2013).

Anthracyclines, including daunorubicin (DNR), which is conventionally used for induction therapy of ALL, are cell cycle non-specific agents that lead to the inhibition of DNA replication and transcription (Chung and Youn, 2016). The exact mechanism of anthracycline-induced cardiotoxicity is still not fully understood despite extensive research. The most widely accepted mechanism is the generation of reactive oxygen species (ROS), which are related to oxidative stress. During the metabolism of anthracyclines, unpaired electrons can be transferred to oxygen molecules to form superoxide radicals, which can cause cellular damage by the degradation of the sarcomere, mitochondrial dysfunction, and DNA damage (Salazar-Mendiguchía et al., 2014). The histological pathophysiology of anthracycline-induced cardiotoxicity is characterized by myocardial damage due to proteolysis, necrosis, apoptosis, and fibrosis (Chung and Youn, 2016).

The literature on anthracycline-associated cardiotoxicity has been focused on late effects among cancer survivors, and it has also been demonstrated that survivors would experience increased risks for cardiomyopathy and cardiovascular disease, which are the leading cause of non-relapse mortality (Oeffinger and Hudson, 2004; Franco et al., 2011; Lipshultz et al., 2013). In general, there is a long latency period between anthracycline exposure and symptomatic cardiotoxicity, and cardiotoxicity is difficult to clinically observe in its early stages. Once it is symptomatic, there is often limited time left to try different treatment strategies, resulting in short-lived outcomes (Leisenring et al., 2009; Robison et al., 2009; Armstrong and Ross, 2014). There is currently no consensus on the best management or treatment options for anthracycline-related cardiotoxicity. Therefore, it is very important to dynamically monitor the earliest cardiac changes in cardiac structure and function and explore timely intervention in children with ALL. In our medical centre, it is routine to monitor the cardiac changes during chemotherapy. To understand the early-onset cardiac disorders after administration of DNR, we retrospectively analysed the clinical features, laboratory examinations and outcomes of cardiac disorders during the first year of chemotherapy in pediatric ALL patients.

## Methods

### Study Design and Participants

This retrospective cohort study included inpatients from Wuhan Union Hospital from January 1, 2015 to December 31, 2018. All patients were diagnosed with ALL and treated according to the Chinese Children’s Cancer Group ALL 2015 (CCCG-ALL-2015) protocol (Cai et al., 2019). The clinical outcomes of cardiac disorders were monitored up to December 31, 2019. Exclusion criteria included lack of information about cardiac parameters, pre-existing cardiovascular diseases or abnormal baseline electrocardiogram (ECG) or echocardiography (Echo). Ethical approval for this study was granted by the Wuhan Union Hospital Human Research Ethics Committee (approval number, 2016108EP). All patients provided informed consent prior to participation.

### Chemotherapy Protocol

**FIGURE 1:**
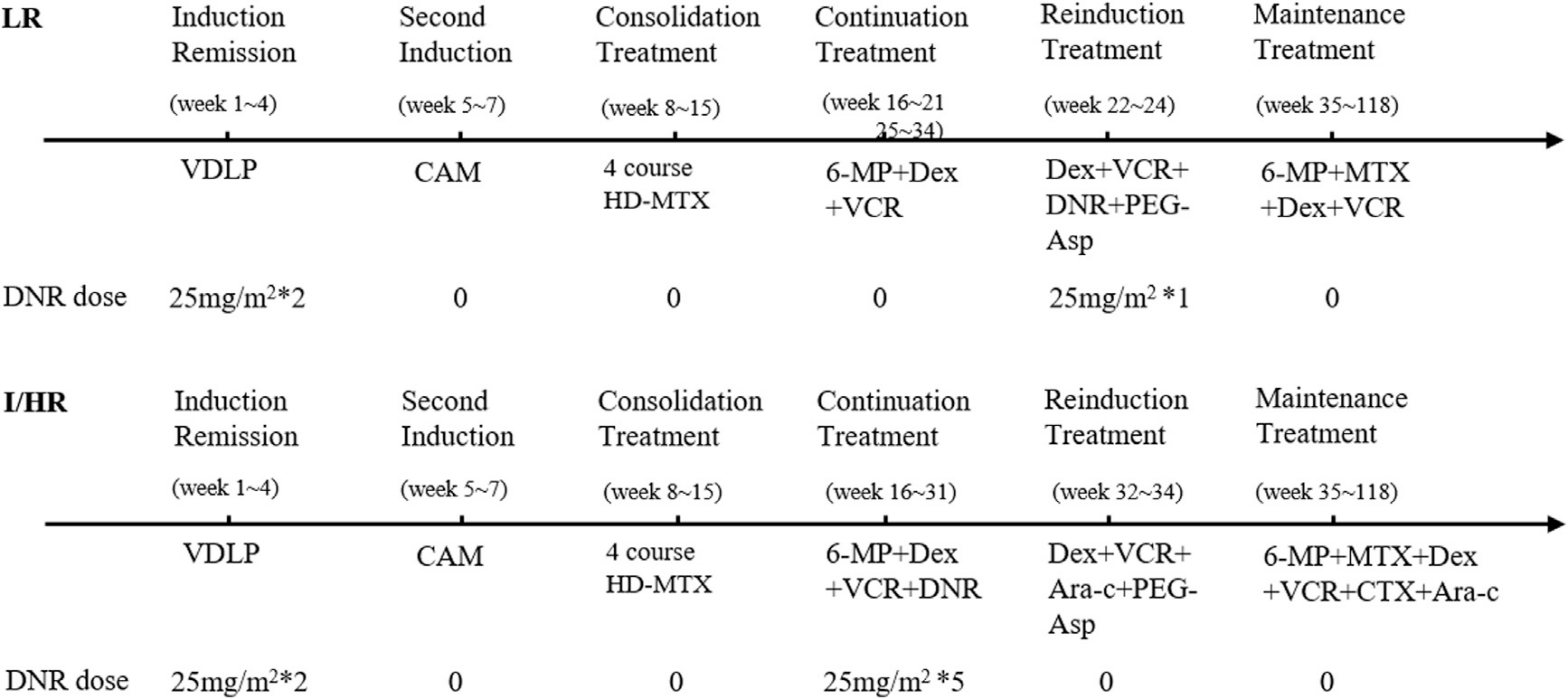
The detailed information of DNR administration in CCCG-ALL-2015 protocol. DNR. daunorubicin; LR, low risk; I/HR, intermediate/high risk; VDLP, vincristine (VCR), daunorubicin (DNR), pegaspargase (PEG-Asp) and prednisone (P); CAM, cyclophosphamide (CTX), cytarabine (Ara-C) and mercaptopurine (6-MP); HD-MTX, high dose of methotrexate; Dex, dexamethasone.

The CCCG-ALL-2015 protocol consists of an induction remission cycle with DNR 25 mg/m^2^ on days 5 and 12; vincristine (VCR) 1.5 mg/m^2^ on days 5, 12, 19, and 26; and pegaspargase (Peg-Asp) 2000 U/m^2^ on days 6 and 26. A second induction cycle with cyclophosphamide (CTX) 1000 mg/m^2^ on day 29 and cytarabine (Ara-C) 50 mg/m^2^ every 12 h on days 29–35. Then, consolidation treatment consisted of four cycles with high-dose methotrexate (HD-MTX) 3–5 g/m^2^ every 2 weeks. Continuation treatment included VCR 1.5 mg/m^2^ for the low-risk (LR) group, while VCR 1.5 mg/m^2^ and five cycles of DNR 25 mg/m^2^ were used for the intermediate/high-risk (I/HR) group. Reinduction treatment included VCR 1.5 mg/m^2^, DNR 25 mg/m^2^ and Peg-Asp 2000 U/m^2^ for the LR group, while VCR 1.5 mg/m^2^ and Peg-Asp 2000 U/m^2^ were used for the I/HR group. Maintenance treatment included oral administration of 6-mercaptopurine (6-MP) and methotrexate (MTX) tablets. The detailed course of chemotherapy protocol is shown in Figure 1.

### Data Collection and Definition

We collected the data including demographic information, risk stratification, cumulative dose of DNR, clinical manifestations and laboratory tests related to heart disorders from electronic medical records. Cardiac disorders were defined according to Common Terminology Criteria for Adverse Events (CTCAE) Version 5.0 (U.S. Department of Health and Human Serivces, National Institutes of Health, National Cancer Institute, 2017). Routine cardiac examinations included ECG and Echo, which were scheduled at the following times, before the start of induction, consolidation, continuation, reinduction and maintenance courses. Serum creatine kinase-MB (CK-MB), troponin T (cTn), brain natriuretic peptide (BNP), cardiac magnetic resonance imaging (CMRI) or cardiac emitted single optical quantum computed tomography (ECT) were arranged for the patients with typical clinical manifestations. Meanwhile, the time of onset and recovery of cardiac disorders were recorded. Patients with obvious cardiac symptoms or signs after administration of DNR were summarized as cases in detail. All data were checked and the diagnosis of cardiac disorders was made by two physicians (HY and YQ) and a third researcher (FZ) adjudicated any difference in interpretation between the two primary reviewers.

### Detection of Polymorphisms of SLC28A3 and UGT1A6

Polymorphisms of metabolic genes of anthracyclines were detected in 23 patients by the pyrosequencing assay, including the solute carrier family 28, member 3 (SLC28A3) and UDP-glucuronyl transferase family 1A, isoform 6 (UGT1A6) (Nagasawa et al., 2001; Aminkeng et al., 2016; Eduardo et al., 2018). The pyrosequencing assay consisted of primer design, PCR amplification, single-stranded sequencing template preparation and sequencing. A set of pyrosequencing primers included a pair of PCR primers, one of which is modified with biotin group at the 5′ end and a separate sequencing primer (All the primers were designed by using the Pyromark Assay as shown in Table 1). PCR amplification was carried out in a 25 μl reaction volume, which contained HsTaq Buffer (TaKaRa), dNTPs (TaKaRa), PCR primers, Hotstart Taq (TaKaRa), genomic DNA and ddH_2_O. Thermocycling was programmed as follows: pre-denaturation at 95°C for 5 min, 50 cycles of 95°C for 20 s, 60°C for 20 s, 72°C for 20 s and final extension at 72°C for 7 min. PCR products binding with the streptavidin Sepharose HP beads (GE) were isolated by the purification tubes (Wuhan First Biotech) and denatured by alkaline solution to gain a single-strand sequencing template, which was pyrosequenced by PYROSEQ-E16 (Wuhan First Biotech) after adding annealing buffer and sequencing-primer and pyrosequencing the enzyme mix and substrates (Qiagen).

**TABLE 1:** The pyrosequencing primer-set of SLC28A3 and UGT1A6.

Gene	SNP	Prime
SLC28A3	rs7853758	PCR forward primer:
		5′-CAA​ACC​AGG​ACA​GGG​CTG​AA-3′
		PCR reverse primer:
		5′-biotin-CCTCCTCCATCTCCCTGGTG -3′
		Sequencing primer:
		5′-TTG​CCT​TCC​TGG​CCC​TG-3′
UGT1A6	rs17863783	PCR forward primer:
		5′-biotin-CAGGTGCTACACAAAGTTTTCAGAC-3′
		PCR reverse primer:
		5′-AAC​AGA​CAA​TAA​AAT​AGA​TAG​GGC​TCC-3′
		Sequencing primer:
		5′-ATGACTTTTT CCCAACGAGT-3′

### Statistical Analysis

Continuous variables are expressed as the mean and standard deviation (SD) or median and interquartile range (IQR). Categorical variables are presented as frequency rates and percentages and were analysed by using the *χ*2 test or Fisher’s exact test as appropriate. Binary logistic regression analysis was used to identify risk factors associated with cardiac disorders. Statistical analysis was performed by using the Statistical Package for Social Sciences version 20.0 software (SPSS Inc., Chicago, IL, United States). A *p* value of <0.05 is considered statistically significant.

## Results

**FIGURE 2:**
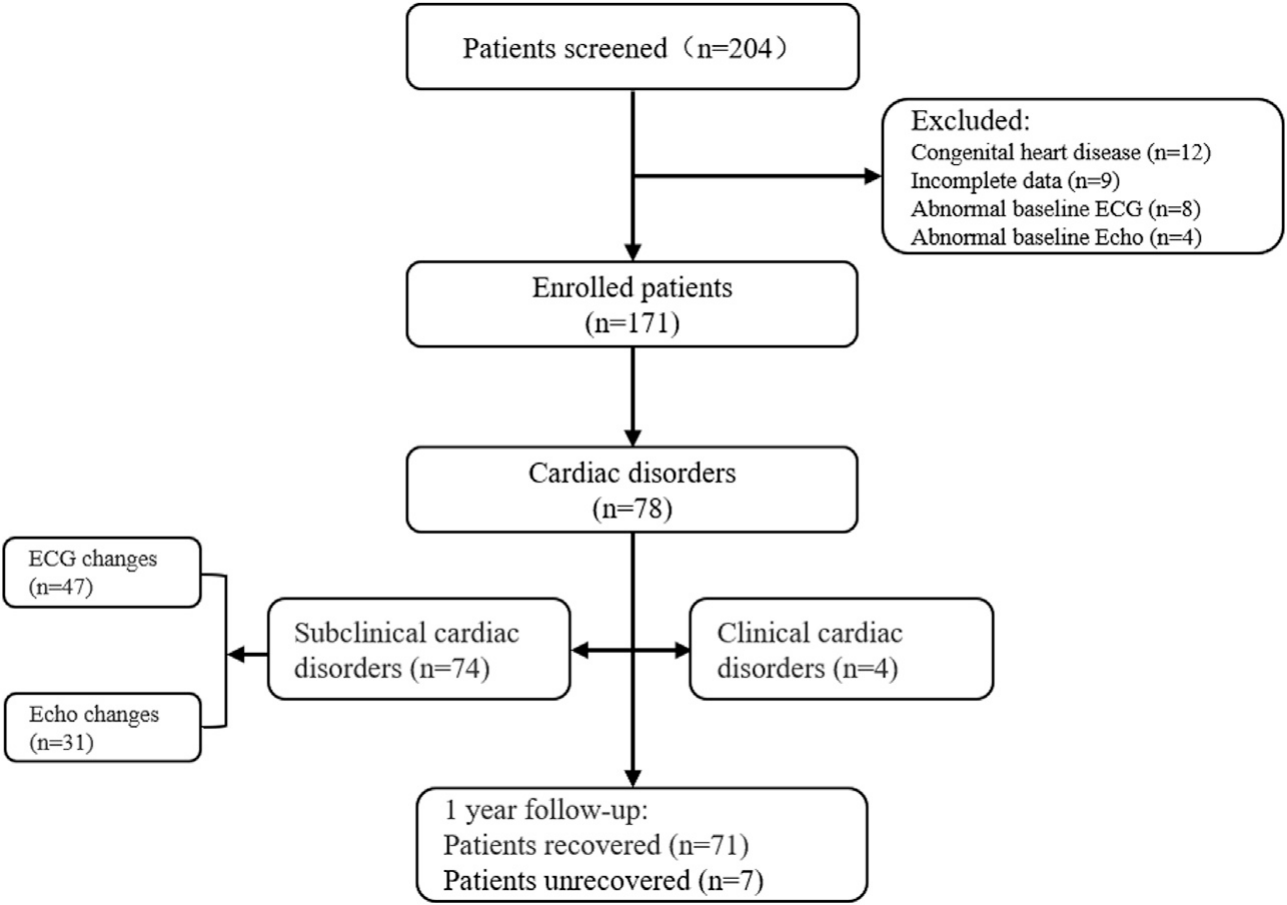
Patients enrolment flow chart.

A total of 204 patients were diagnosed with ALL; 33 patients (16.18%) were excluded for lack of information about cardiac parameters (9, 4.41%) and for congenital heart disease or abnormal baseline ECG/Echo at diagnosis (24, 11.76%). This study included 171 eligible patients. Patients enrolment flow chart is shown in Figure 2. The median age was 5.07 ± 3.19 years (IQR, 3–7; range 7 months–14 years), and 108 patients (63.16%) were boys. The characteristics of the 171 ALL patients and incidence of cardiac disorders are shown in Table 2. The incidence of cardiac disorders was not associated with age, sex or risk classification (*p* > 0.05) but with cumulative DNR dosage (*p* = 0.030, OR = 1.553, 95% CI: 1.005–3.108) (Table 3).

**TABLE 2:** Characteristics of the ALL patients and the incidence of cardiac disorders.

Characteristics	Covariable distribution (n/%)		Cardiac disorders incidence (n/%)		*p*
Age at diagnosis					0.786
0–1 year	16	(9.36%)	5	(31.25%)	
2–10 years	134	(78.36%)	59	(44.03%)	
≥10 years	21	(12.28%)	10	(47.62%)	
Sex					0.882
Female	63	(36.84%)	28	(44.44%)	
Male	108	(63.16%)	46	(42.59%)	
Risk stratification					0.896
Low risk	91	(53.22%)	40	(43.96%)	
Intermediate risk	68	(39.77%)	30	(44.12%)	
High risk	12	(7.01%)	4	(33.33%)	
Cumulative DNR dosage					**0.027**
≤75 mg/m^2^	84	(49.12%)	25	(29,76%)	
>75 mg/m^2^	87	(50.88%)	49	(56.32%)	

Bold values indicates of the p value of <0.05

**TABLE 3:** Binary logistic regression analysis to identify risk factors associated with cardiac disorders.

Covariable		B	S.E	Wald	Sig.	Odds ratio with 95% CI
Age	0–1 year					
	2–10 years	1.135	0.714	2.525	0.112	3.112 (0.767–12.621)
	≥10 years	0.309	0.523	0.349	0.554	1.362 (0.489–3.793)
Sex	Female					
	Male	−0.207	0.334	0.386	0.534	0.813 (0.422–1.563)
Risk stratification	LR					
	I/H	1.219	0.683	3.184	0.074	3.384 (1.887–8.910)
Cumulative DNR dosage	≤75 mg/m^2^					
	>75 mg/m^2^	−1.805	0.705	6.555	**0.030**	1.553 (1.005–3.108)

Bold values indicates of the p value of <0.05

**FIGURE 3:**
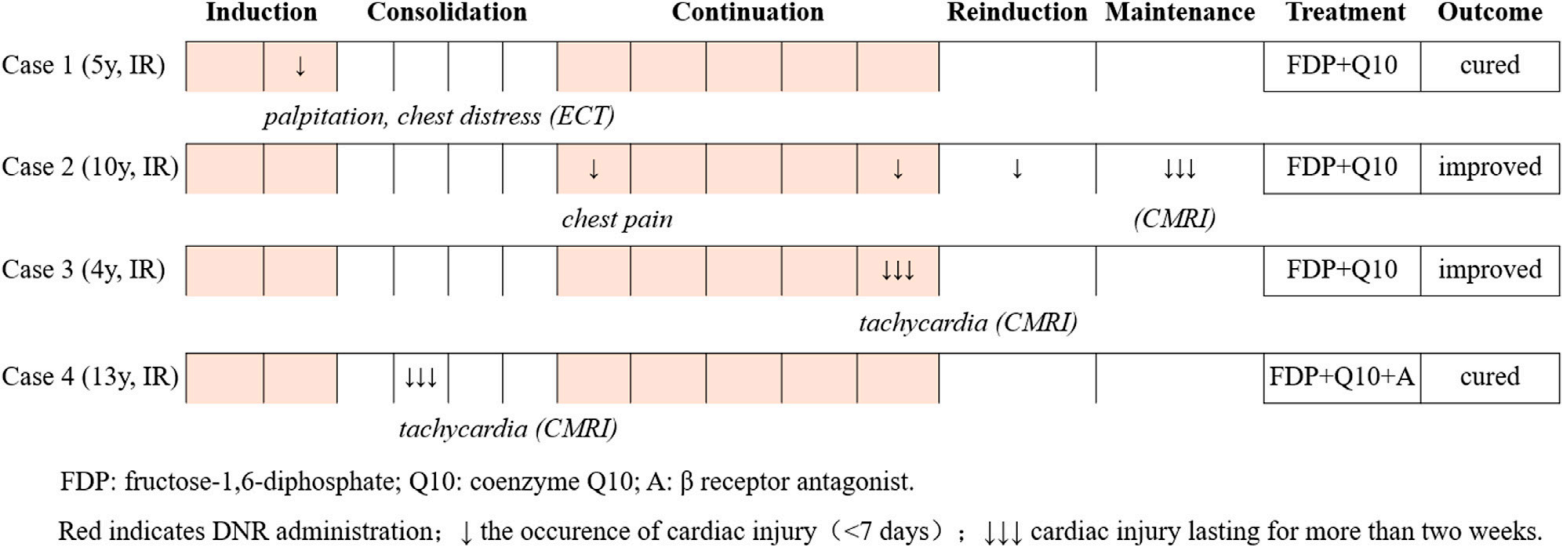
Clinical features and outcomes of patients with cardiac disorders after DNR administration.

**FIGURE 4:**
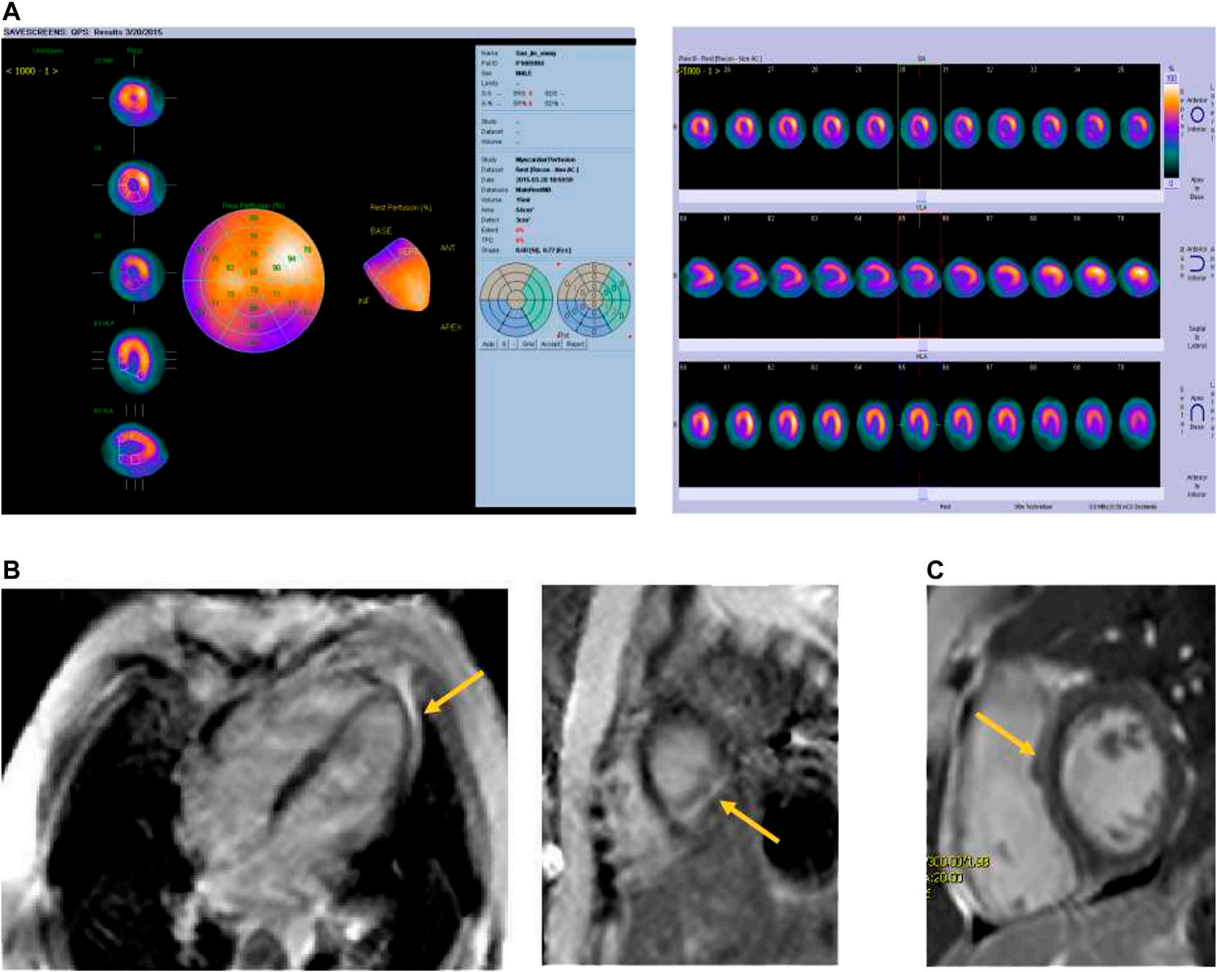
The images of cardiac disorders of ALL patients. **(A)** Cardiac ECT showed myocardial ischaemia, a significant decrease in blood perfusion in the posterior left ventricular wall; **(B)** Cardiac Late Gd imaging in horizontal long-axis and short axis demonstrated myocardial enhancement in the inferior left ventricular wall and apex; **(C)** Cardiac Late Gd imaging in short axis demonstrated a midwall enhancement on the ventricular septum.

Four patients (2.34%) suffered from clinical cardiotoxicity after administration of DNR (Figure 3). Case 1 complained of palpitation and chest distress after the second administration of DNR during induction remission. The results of serum biomarkers (CK-MB, cTn and BNP) and Echo were normal. However, the ECG showed sinus tachycardia and abnormal T-waves. Cardiac ECT showed myocardial ischaemia with a significant decrease in blood perfusion in the posterior left ventricular (LV) wall (Figure 4A). Case 2 initially presented with chest pain during continuation treatment with normal serum biomarkers and Echo. The chest pain lasted intermittently for 8 months, and CMRI showed myocardial damage and a small amount of pericardial effusion after completing reinduction treatment (Figure 4B). Case 3 and case 4 were found to have tachycardia lasting for more than one month by physical examination. The CMRI of case 3 showed myocarditis (Figure 4C) and that of case 4 was normal.

**FIGURE 5:**
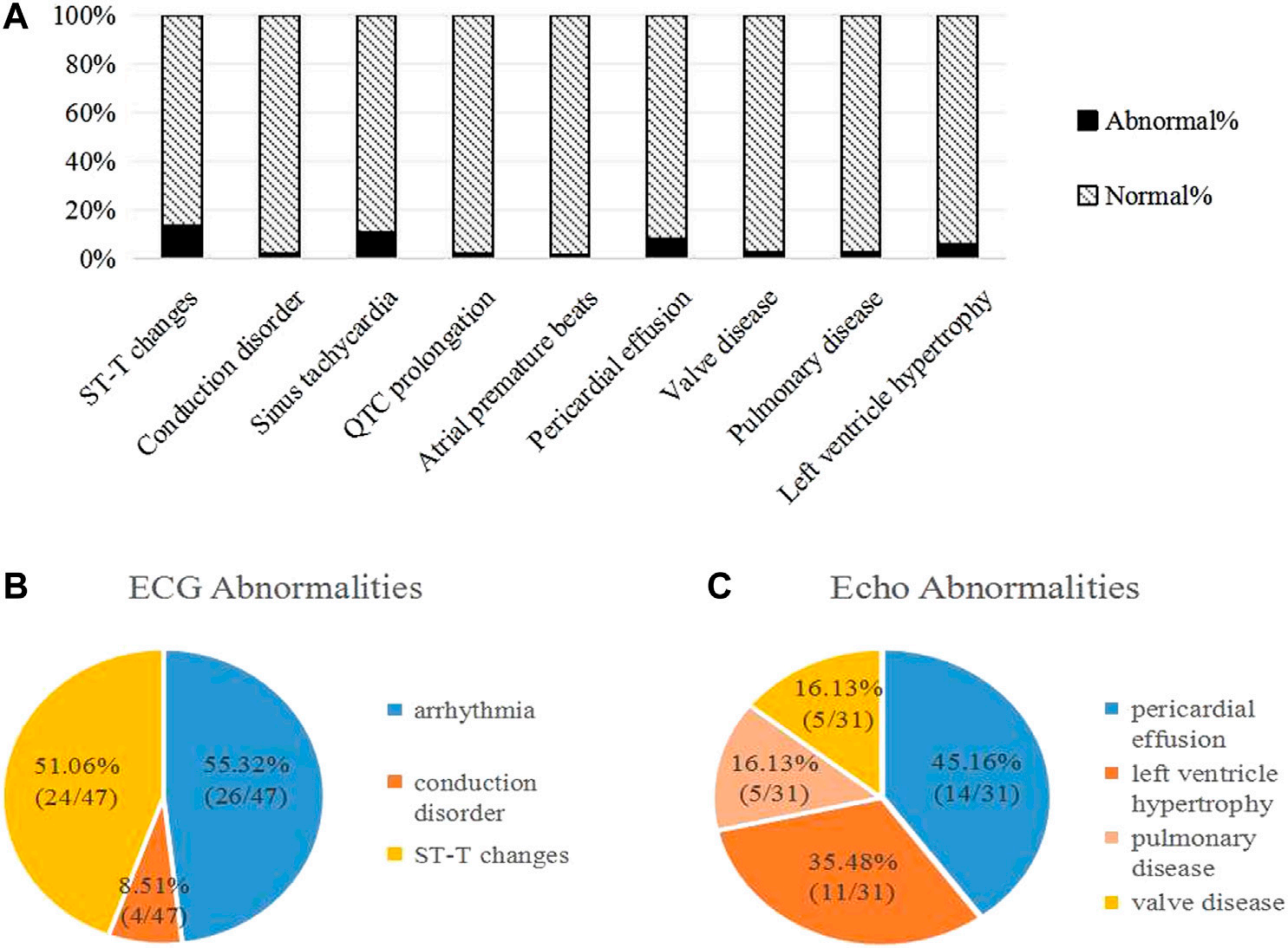
Abnormalities of subclinical cardiac disorders in ALL children during the first year of chemotherapy. **(A)** Abnormalities on ECG and Echo; **(B)** ECG Abnormalities; **(C)** Echo Abnormalities. Some patients had more than one of above changes on ECG/Echo.

A total of 74 patients (43.27%) were found to have developed subclinical cardiac disorders by regular cardiac examinations (Figure 5A). ECG abnormalities were found in 47 children (27.49%), including arrhythmias (26/47, 55.32%), conduction disorders (4/47, 8.51%) and nonspecific ST-T changes (24/47, 51.06%) (Figure 5B). There were several types of arrhythmias, such as sinus tachycardia, QTc prolongation and atrial premature beats. Echo abnormalities were found in 31 patients (18.13%) (Figure 5C). There were 14 cases (14/31, 45.16%) with a small amount of pericardial effusion, 11 cases (11/31, 35.48%) with LV hypertrophy, 5 cases (5/31, 16.13%) with a widened pulmonary artery and 5 cases (5/31, 16.13%) with valve disease. One patient with pulmonary regurgitation showed a progressive trend from a widened pulmonary artery to pulmonary hypertension, without significant decline in left ventricular ejection fraction (LVEF).

Among these patients with subclinical cardiac disorders (Table 4), ECG changes were commonly seen during the course of induction treatment (25/171, 14.59%) and continuation treatment (17/171, 9.90%). Most of the ECG abnormalities reversed without intervention, with a median time of 7.93 ± 1.03 weeks. There were six ECG abnormalities (6/171, 3.49%) occurring in the course of consolidation treatment, which recovered with a median time of 4.67 ± 2.45 weeks. Echo abnormalities, including pericardial effusion, widened pulmonary artery, valve disease and LV hypertrophy, mainly occurred after the course of induction treatment (27/171, 15.70%), and most of them self-reversed within 10 weeks.

**TABLE 4:** Subclinical cardiac disorders overt the course of 1-year on-protocol therapy.

Description	Treatment course						
	Induction remission	Second induction	Consolidation treatment	Continuation treatment	Reinduction treatment	Maintenance treatment	One-year after treatment
Abnormal ECG							
Conduction disorder	1 (0.58%)	1 (0.58%)	—	2 (1.16%)	—	—	2 (1.16%)
Sinus tachycardia	8 (4.68%)	2 (1.16%)	2 (1.16%)	5 (2.92%)	2 (1.16%)	1 (0.58%)	1 (0.58%)
ST-T changes	7 (4.09%)	5 (2.92%)	3 (1.75%)	6 (3.49%)	3 (1.75%)	—	1 (0.58%)
QTc prolongation	—	—	1 (0.58%)	3 (1.75%)	—	—	1 (0.58%)
Atrial premature beats	—	1 (0.58%)	—	1 (0.58%)	—	—	—
Total	16 (9.35%)	9 (5.24%)	6 (3.49%)	17 (9.90%)	5 (2.92%)	1 (0.58%)	5 (2.92%)
Abnormal Echo							
Pericardial effusion	—	5 (2.92%)	6 (3.49%)	3 (1.75%)	—	—	1 (0.58%)
Pulmonary disease	—	2 (1.16%)	1 (0.58%)	2 (1.16%)	—	—	1 (0.58%)
Valve disease	—	—	2 (1.16%)	3 (1.75%)	—	—	—
Left ventricle hypertrophy	1 (0.58%)	—	4 (2.32%)	6 (3.49%)	—	—	—
Total	1 (0.58%)	7 (4.08%)	13 (7.55%)	14 (8.15%)			2 (1.16%)

The patients with cardiac disorders recovered in one year after the onset, except 7 children (7/171, 8.97%). The detailed information about the seven unrecovered patients is summarized in Table 5. The cardiac abnormalities included incomplete atrioventricular block (2 cases), ST-T changes (1 case), QTc prolongation (1 case), sinus tachycardia (1 case), pericardial effusion (1 case) and pulmonary arterial hypertension (1 case). The genotypes of the UGT1A6 and SLC28A3 genes were detected in 23 patients with cardiac disorders. There were 3 cases (3/7, 42.85%) with UGT1A6 GT genotype (indicating a high risk of cardiotoxicity) in the unrecovered group, which was higher than that in the recovered group (2/16, 12.50%) (*χ*2 = 1.500, *p* = 0.221). The frequency of the SLC28A3 GA genotype (indicating a low risk of cardiotoxicity) was similar in the two groups (unrecovered: 1/7, 14.29% vs. recovered: 3/16, 18.75%, *χ*2 = 0.539, *p* = 0.463).

**TABLE 5:** Clinical characteristics of the unrecovered patients. The unrecovered parameters are marked with an asterisk (*). ECG, electrocardiogram; Echo, echocardiography; DNR, daunorubicin; M, male; F, female; LR, low risk; IR, intermediate risk; IRBBB, incomplete right bundle branch block; PAH, pulmonary arterial hypertension.

No.	Age (year)	Risk	Occurrence course	ECG	Echo	Biochemical markers	Genes	DNR (mg/m^2^)
1	1–10	IR	Consolidation treatment	ST-T changes	*Pericardial effusion	Normal	**SLC28A3:GA**	175
							UGT1A6:GG	
2	1–10	LR	Continuation treatment	ST-T changes	*PAH	Normal	SLC28A3:GG	75
							**UGT1A6:GT**	
3	1–10	LR	Induction remission	*IRBBB	Normal	Normal	SLC28A3:GG	75
							**UGT1A6:GT**	
4	1–10	LR	Second induction	*T wave changes	Normal	Normal	SLC28A3:GG	75
							**UGT1A6:GT**	
5	10–14	IR	Reinduction treatment	*QTc prolongation	Normal	Normal	SLC28A3:GG	175
							UGT1A6:GG	
6	1–10	LR	Consolidation treatment	*IRBBB	Normal	Normal	SLC28A3:GG	75
							UGT1A6:GG	
7	1–10	IR	Continuation treatment	*Sinus tachycardia	Normal	Normal	SLC28A3:GG	175
							UGT1A6:GG	

Bold values indicates of the SLC28A3 GA genotype (indicating a low risk of cardiotoxicity) and UGT1A6 GT genotype (indicating a high risk of cardiotoxicity).

## Discussion

Recently, there have been many studies focusing on the long-term cardiotoxicity of childhood cancer survivors. Traditionally, anthracycline-induced cardiotoxicity is defined as a >10% decline in LVEF and drop to <50% (Chinese Society of Clinical Oncology, Chinese Sopciety of Hematology, 2013). The cardiotoxicity characterized by the decline of LVEF usually presents as arrhythmia, pericarditis, HF and cardiomyopathy (Chinese Society of Clinical Oncology, Chinese Sopciety of Hematology, 2013). However, the treatment-related cardiotoxicity during the on-protocol period is poorly studied. In our study, the on-protocol cardiotoxicity with LVEF reduction was not observed. We found a high incidence of cardiac disorders (45.61%) which is higher than the 3–26% reported in the literature (Joerg et al., 2014). Reported frequencies of cardiotoxicity with lower doses have varied widely, owing to studies in different patient populations with differing definitions of cardiotoxicity and varying periods of follow-up (Henriksen, 2017). However, it is mentioned that regular ECG and Echo could show paediatricians the early changes in cardiac function or structure.

Our study suggested that Grade 1 and 2 cardiac disorders are commonly seen in children with ALL during the chemotherapy. Most of the Grade 1 cardiac events with alterations in electrophysiological or cardiac imaging were identified by routine ECG and Echo monitoring, which generally self-remitted after 2 months. A few of the cardiac changes, such as arrhythmia, LV enlargement, and pulmonary valve stenosis, were sustainable and even aggravating. Zhong et al. (Wang, 1991) reported that Grade 1 cardiac disorders shown on ECG were found in 24.5% patients who received adriamycin. The abnormal ECGs included arrhythmias (11 cases), non-specific ST-T changes (12 cases) and low voltage (14 cases). Most patients were cured, but 4 cases developed fatal CHF (Wang, 1991). We found that Grade 2 or higher events were rare, including obvious chest pain, palpitations, and persistent tachycardia. These symptoms or signs could occur in different treatment courses without LVEF decline. Myocardial injury could be confirmed by CMRI. To our acknowledge, the Grade 2 or higher cardiac events reported are characterized by reduced LVEF, cardiomyopathy or symptomatic CHF, which generally occur years after chemotherapy. It indicates that we may greatly underestimate the incidence of cardiac events if we define cardiotoxicity as LVEF decline.

Although we found that the clinical manifestations could be reversed by intensive treatment for myocardial damage, whether the early-onset cardiac disorders develop into LV dysfunction in the future remains to be followed up for a long time. Hyun et al. found enlarged LV size, pulmonary hypertension and decreased LVEF were independent predictors of non-recovery of LV dysfunction in breast cancer patients (Yoon et al., 2019). Therefore, cardiac monitoring was strongly recommended not only during the off-protocol period but also during the on-protocol period. In addition, our data showed the course-specific prevalence of cardiac disorders in children with ALL. The highest course-specific prevalence occurred after induction and continuation treatments. Peter et al. reported that the median time of developing cardiotoxicity after anthracycline exposure was 3.5 months, which is consistent with our result (Henriksen, 2017). These finding suggested that a routine evaluation of cardiac function should be carried out after a course of anthracycline exposure.

The predictors of incident early cardiotoxicity are not clear. As shown in the study of late-onset cardiac dysfunction in childhood cancer survivors, a higher cumulative anthracycline dose and a younger age at diagnosis are suggested to be risk factors (Von Hoff et al., 1979; Lipshultz and Adams, 2010). In this study, we observed a relation between cumulative DNR dosage and cardiac disorders, but no obvious relation between the incidence and the age, sex and risk stratification. Chemotherapy dosage in the CCCG-ALL-2015 protocol was based on body surface area, and the cumulative dosage of DNR was 175 mg/m^2^, which was well below the maximum dose (500 mg/m^2^). Lipshultz et al. reported that subclinical cardiotoxicity occurred in 30% of patients with a cumulative dose of 180–240 mg/m^2^ anthracyclines, presenting as an asymptomatic decrease in LV function (Lipshultz and Adams, 2010). Cardiac injury can occur even at anthracycline doses below 100 mg/m^2^ (Lipshultz and Adams, 2010; Pal et al., 2010). It is indicated that there is no absolute safe dosage of anthracycline drugs. On the other hand, few patients suffered from severe HF and cardiomyopathy after chemotherapy (Broder et al., 2008; Cuomo et al., 2019). The reason may be related to the genetic variability, which affects enzymatic activity involved in absorption, distribution metabolism and excretion of anthracyclines (Nagasawa et al., 2001; Aminkeng et al., 2016; Eduardo et al., 2018).

The intake of anthracyclines in tissues and leukemia cells depends on gene variations in the influx and efflux transporters (Nagasawa et al., 2001; Aminkeng et al., 2016). The solute carrier super family (SLC) are influx transporters of anthracyclines. Two variants (rs7853758 and rs885004) in SLC28A3 have been shown to have associations with anthracycline-induced cardiotoxicity in three independent pediatric cohorts, with the A-allele (rs7853758) of the variant conferring a reduced cardiotoxicity risk (Visscher et al., 2012; Visscher et al., 2013; Aminkeng et al., 2016). UGT1A6 plays a role in the drug detoxi?cation glucuronidation pathway and reduced UGT1A6-mediated glucuronidation of anthracycline metabolites may lead to the accumulation of toxic metabolites in patients, resulting in an increased risk of developing cardiotoxicity (Visscher et al., 2012; Visscher et al., 2013). It has been reported that the UGT1A6 variant (T-allele) is associated with a significantly increased risk of cardiotoxicity in childhood cancer survivors (*p* = 0.0062, OR = 7.98, 95% CI: 1.85–34.4) (Visscher et al., 2012; Visscher et al., 2013). To explore the role of genetic variability in the prediction of early cardiotoxicity, we detected and found that the UGT1A6 and SLC28A3 variants were not correlated with cardiac disorders (*p* > 0.05). This may be related to the small sample of patients. In the future, large-scale clinical studies are needed to validate the correlation between cardiotoxicity and polymorphisms of metabolic genes of anthracyclines. Based on pharmacogenomic testing, we can further develop individualized chemotherapy for cancer patients.

In general, symptomatic cardiotoxicity induced by anthracyclines is serious with a poor prognosis, and early interventions and treatments can greatly improve the outcomes of cancer patients (Jensen et al., 2011; Ky et al., 2014). There are some recommendations for interventions, including critically controlling the cumulative dosage of anthracyclines (Chinese Society of Clinical Oncology, Chinese Society of Hematalogy, 2013); using dexrazoxane (Lipshultz et al., 2014); using liposomal anthracyclines (instead of regular anthracyclines) (Gabizon, 2001; Smith et al., 2010; Henriksen, 2017); and using angiotensin converting enzyme inhibitors (ACEIs), angiotensin receptor blockers (ARBs), β-blockers and statins to prevent and treat HF (Huelsenbeck et al., 2011; Bloom et al., 2016). It is reported that early initiation of standard medical treatment for HF with renin angiotensin inhibitors and β-blockers may lead to LV functional recovery in anthracycline-induced cardiotoxicity. LV systolic dysfunction recovered among 67.3% of patients with a median time of 4 months with the introduction of standard medical treatment for HF (Ohtani et al., 2019). Jensen et al. conducted a prospective, blinded, follow-up observational study in 120 cases with advanced breast cancer and found that, when patients experienced reduced LVEF after chemotherapy, they did not spontaneously regain cardiac function, whereas continued therapy with ACEIs caused a remarkable and long-lasting recovery after more than 3 months (Jensen et al., 2011). In our study, four children with clinical cardiotoxicity received intensive treatment, including coenzyme Q10 and β-blockers, and the clinical manifestations subsequently disappeared.

This study has some limitations. First, due to the nature of retrospective study, some cases were excluded because of incomplete documentation of laboratory testing. It could be better if all unrecovered patients had laboratory findings of CMRI. Since a small number of children had genetic findings, further studies are needed to confirm the correlation between cardiotoxicity and polymorphisms of metabolic genes of anthracyclines. Second, the intervention of the patients with clinical cardiotoxicity was not uniform and planned. We could not make any solid conclusions on the treatment of those patients, and a prospective study is needed to identify proper intervention strategies in the future. Third, the study provided preliminary data of cardiac disorders in on-protocol pediatric ALL patients. However, the follow-up time was not long enough, making it difficult to assess the relation between early cardiac injury and late cardiotoxicity. Our findings need to be validated in a powered clinical study prospectively.

## Conclusion

In conclusion, subclinical cardiac disorders are more common during pediatric ALL chemotherapy than reported in the literature. Regular ECG and Echo, combined with CMRI when necessary, can help physicians to identify patients with asymptomatic cardiac disorders. Thus, efforts to improve outcomes for children with ALL should include prevention and mitigation of on-protocol cardiotoxicity.

## Data Availability

The raw data supporting the conclusion of this article will be made available by the authors, without undue reservation.
